# The interplay between psychopathological symptoms: transdiagnostic cross-lagged panel network model

**DOI:** 10.1192/bjo.2022.516

**Published:** 2022-06-27

**Authors:** UnYoung Chavez-Baldini, Karin Verweij, Derek de Beurs, Claudi Bockting, Anja Lok, Arjen L. Sutterland, Geeske van Rooijen, Guido van Wingen, Damiaan Denys, Nienke Vulink, Dorien Nieman

**Affiliations:** Department of Psychiatry, Amsterdam University Medical Center, University of Amsterdam, The Netherlands; Department of Psychiatry, Amsterdam University Medical Center, University of Amsterdam, The Netherlands; Department of Epidemiology, Netherlands Institute of Mental Health and Addiction (Trimbos Institute), The Netherlands; Department of Psychiatry, Amsterdam University Medical Center, University of Amsterdam, The Netherlands; Department of Psychiatry, Amsterdam University Medical Center, University of Amsterdam, The Netherlands; Department of Psychiatry, Amsterdam University Medical Center, University of Amsterdam, The Netherlands; Department of Psychiatry, Amsterdam University Medical Center, University of Amsterdam, The Netherlands; Department of Psychiatry, Amsterdam University Medical Center, University of Amsterdam, The Netherlands; Department of Psychiatry, Amsterdam University Medical Center, University of Amsterdam, The Netherlands; Department of Psychiatry, Amsterdam University Medical Center, University of Amsterdam, The Netherlands; Department of Psychiatry, Amsterdam University Medical Center, University of Amsterdam, The Netherlands

**Keywords:** Transdiagnostic, longitudinal, network analysis, psychopathology, symptom network

## Abstract

**Background:**

Recent paradigm shifts suggest that psychopathology manifests through dynamic interactions between individual symptoms.

**Aims:**

To investigate the longitudinal relationships between symptoms in a transdiagnostic sample of patients with psychiatric disorders.

**Method:**

A two-wave, cross-lagged panel network model of 15 nodes representing symptoms of depression, (social) anxiety and attenuated psychotic symptoms was estimated, using baseline and 1-year follow-up data of 222 individuals with psychiatric disorders. Centrality indices were calculated to determine important predictors and outcomes.

**Results:**

Our results demonstrated that the strongest relationships in the network were between (a) more suicidal ideation predicting more negative self-view, and (b) autoregressive relationships of social anxiety symptoms positively reinforcing themselves. Negative self-view was the most predictable node in the network as it had the highest ‘in-expected influence’ centrality, and may be an important transdiagnostic outcome symptom.

**Conclusions:**

The results give insight into longitudinal interactions between symptoms, which interact in ways that do not adhere to broader diagnostic categories. Our results suggest that self-view can also be a transdiagnostic outcome of psychopathology rather than just a predictor, as is normally posited, and may especially have an important relationship with suicidal ideation. Overall, our study demonstrates the dynamic complexity of psychopathology, and further supports the importance of investigating symptom interactions of different psychopathological dimensions over time and across disorders.

The conceptualisation of psychopathology has shifted over the past decade to a dynamic systems perspective, in which a disorder is the result of the dynamic interactions between various mechanisms.^1,2^ This dynamic systems perspective is embodied in the network approach, which posits that psychopathology arises from a network of symptoms that interact over time.^3^ Although the dynamic systems perspective and network approach have gained more traction, there is still little research investigating psychopathology as a transdiagnostic dynamic system, which acknowledges that symptoms can cut across diagnoses. A study assessing the network structure of symptoms from 12 DSM-IV diagnoses in a community sample found that some symptoms of one disorder were also connected to symptoms of different disorders.^4^ Networks with a variety of symptom across various disorders should therefore be further investigated. Additionally, to investigate the dynamic nature of psychopathology, it is important to move from cross-sectional to longitudinal network designs, such as temporal, contemporaneous or cross-lagged panel networks (CLPNs).^5,6^ This could give insight into the interplay of psychopathological dimensions over time at the symptom level by elucidating how observations at one time point predict observations at the next time point, and into transdiagnostic mechanisms by identifying symptoms that play a predictive or influential role in the network.^7^ This could also elucidate important symptom interactions, which could indicate potential causal relationships and points of intervention to disrupt negative processes.^6^

## Relationships between psychopathological symptoms

Some core symptom dimensions of psychopathology include depression, anxiety and psychotic-like symptoms, which may be considered transdiagnostic. Depressive and anxiety symptoms are often reported in patients with various disorders,^8,9^ and psychotic-like experiences can also occur in patients with non-psychotic disorders.^10^ Previous cross-sectional networks demonstrated relationships between (social) anxiety and depression symptoms,^11,12^ and between psychotic and depression symptoms.^13^ In a cross-sectional network analysis with a transdiagnostic sample, we found that sum scores of depression, (social) anxiety and subclinical psychotic symptom dimensions were all interrelated.^14^ It remains necessary, however, to investigate these relationships over time.

## Study aims

The present study therefore investigated two-wave longitudinal relationships over an average of 12 months, between individual depression, (social) anxiety and attenuated psychotic symptoms in a transdiagnostic sample of patients with various psychiatric disorders. The aim was to investigate how symptoms affect each other over time, and to identify important predictor and outcome symptoms. We modelled the longitudinal relationships between symptoms with a CLPN, and investigated the predictability and influence of each item in the network. It was hypothesised that individual symptoms will interact in ways that do not adhere to broader diagnostic categories.

## Method

### Sample

The sample comprised 222 patients with psychiatric disorders recruited during intakes at the out-patient clinic of the Department of Psychiatry at the Amsterdam University Medical Center (UMC), location Academic Medical Center (AMC), which is an expert centre for misophonia, early psychosis, anxiety and depressive disorders. A total of 1134 patients participated in the first measurement, of which 304 completed the follow-up measurement; 82 were excluded because the follow-up measure was not completed within the appropriate time frame.

Inclusion criteria were age 14–75 years, ability to give informed consent, having a DSM-IV-TR or DSM-V diagnosis, fluent in Dutch and completion of the follow-up measurement within 6–18 months. Exclusion criteria were acute high risk of suicide (i.e. suicidal behaviour requiring immediate and urgent attention), unstable medical disorder, premorbid IQ < 70, history of seizure or clinically significant abnormality of the neurological system.

### Procedure

The Across study is an ongoing, longitudinal research project that collects data on cognitive functioning, psychopathology symptoms and biological parameters (https://osf.io/yhvtb/). The full study procedure is described in Nieman et al.^15^ After an intake at the Department of Psychiatry of the Amsterdam UMC, location AMC, patients were invited to participate in the study after being briefed. The authors assert that all procedures contributing to this work comply with the ethical standards of the relevant national and institutional committees on human experimentation and with the Helsinki Declaration of 1975, as revised in 2008. All procedures involving human patients were approved by the Medical Ethical Review Committee and the Biobank Review Committee of the Amsterdam UMC (General Assessment and Registration number NL55751.018.15). All participants and all parents or guardians of minors provided written informed consent to participate in this study. Participants were able to participate at any point of their clinical trajectory (e.g. before, during or after treatment), and could discontinue participation from the study or parts of the study at any time. For the 1-year follow-up, additional consent was obtained.

Participants filled in questionnaires on psychopathological symptoms on a computer, which took 30 min to 1 h to complete. The current study had a two-wave longitudinal design and used baseline (time point 1) and 1-year follow-up (time point 2) questionnaire data.

### Measures

Psychopathological symptoms included in this study were assessed with the Hamilton Rating Scale for Anxiety (HRSA), the Social Interaction Anxiety Scale (SIAS), the Inventory of Depressive Symptomatology Self-Report (IDS-SR), which are validated and psychometrically-sound questionnaires. Moreover, we administered the Psychiatric Dimensions Questionnaire, which was developed at the Amsterdam UMC.^16^ The HRSA measures the severity of somatic, cognitive and affective symptoms of anxiety.^17^ It consists of 13 items that are rated on a scale of 0 (not present) to 4 (severe). The SIAS assesses anxiety in social interactions and fear of scrutiny by others.^18^ It consists of 20 items and each item is rated on a scale of 0 (not at all characteristic of me) to 4 (extremely characteristic of me). The IDS-SR measures the severity of depressive symptoms pertaining to mood, cognition, arousal, suicidality and sleep.^19^ It consists of 30 items that are rated on a scale from 0 (symptom is not present) to 3 (strongest impairment). The Psychiatric Dimensions Questionnaire consists of 26 items and assesses a variety of transdiagnostic concepts that are commonly affected in patients with a psychiatric disorder: affect, volition, identity, cognition, reality and vitality.^15,16^ Only items from the reality subscale, in which participants rate questions pertaining to attenuated psychotic symptoms (i.e. exceptional experiences and anomalous self-experiences) on a scale of 0 (never) to 8 (continuously), are included in the network. Exceptional experiences and anomalous self-experiences refer to experiential deviations, such as *déjà vu*, inexplicable auditory or visual perceptions, or difficulty in grasping taken-for-granted meanings.^20,21^ Other subscales items were not included because they are covered by the other questionnaires or were not part of the aforementioned core dimensions. Only the psychological items from the questionnaires were included, meaning any somatic or physical symptoms were excluded.

Age, gender, diagnostic category and presence of treatment were included as covariates in the network. Age and gender were obtained from a demographic questionnaire. Diagnostic category and treatment were obtained from the participants’ medical records. The diagnosis is determined by a psychiatrist and categorised into seven categories: schizophrenia spectrum and other psychotic disorders, depressive disorders, anxiety disorders, obsessive–compulsive and related disorders, impulse-control disorder not otherwise specified (misophonia), bipolar disorder and other disorders. Specific diagnoses under each category can be viewed in Supplementary Table 1 available at https://doi.org/10.1192/bjo.2022.516. Presence of treatment was measured with two variables: treatment at time point 1, with 0 indicating no treatment before or during the research phase and 1 indicating treatment was started before time point 1; and treatment between time points 1 and 2, with 0 indicating no treatment before or during the research phase and 1 indicating treatment was started between time points 1 and 2. Treatment included both psychotropic medication use (e.g. antidepressants) and psychological treatment or support (e.g. cognitive–behavioural therapy).

Individual items that were used in the analyses can be viewed in Table 1. Each node represents a single item from a questionnaire, except for three items that were combined, which is indicated in the rightmost column. A two-step item selection procedure was performed before the analyses, using content-based selection as recommended by Rhemtulla et al^22^ and weighted topological overlap approach, which is detailed in Supplementary Appendix 1. This reduced the total number of items from 79 to 15.

**Table 1:** Symptom nodes and labels

Node	Label	Items	Measure (item number)
Sad	Sad	Feeling sad	IDS-SR (5)
Self	Self-view	(Negative) View of myself^a^	IDS-SR (16)
Sui	Suicidal ideation	Thoughts of death or suicide^b^	IDS-SR (18)
Int	Interest	(Lack of) General interest	IDS-SR (19)
EyCon	Difficulty making eye contact	I have difficulty making eye contact with others	SIAS (2)
DifCo	Difficulty mixing with co-workers	I find it difficult to mix comfortably with the people I work with	SIAS (4)
SocT	Social tension	I tense up if I meet an acquaintance in the street; I feel tense if I am alone with just one other person	SIAS (6 + 8)
Talk	Difficulty talking with others	I have difficulty talking with other people	SIAS (10)
Dis	Difficulty disagreeing	I find it difficult to disagree with another's point of view	SIAS (13)
AnxT	Anxious tension	Anxious mood: worries, anticipation of the worst, fearful anticipation, irritability; Tension: feelings of tension, fatigability, startle response, moved to tears easily, trembling, feelings of restlessness, inability to relax	HRSA (1 + 2)
Fear	Fears	Fears: of dark, of strangers, of being left alone, of animals, of traffic, of crowds	HRSA (3)
PerAn	Perceptual anomalies	To what extent can you perceive things that others cannot perceive?	Dimensions (10)
Perplx	Perplexity, lack of natural self-evidence	Do you feel that the natural self-evidence of the world around you has been lost? Do you have the profound experience that you have to think about the most obvious things, such as about everyday actions or objects?	Dimensions (11 + 12)
AbSal	Aberrant salience	Has your perception changed, making everything more meaningful?	Dimensions (13)
Threat	Feeling threatened or paranoid	Do you feel that others want to harm you?	Dimensions (14)

In the ‘Measure (item number)’ column, the questionnaire and the item number that each node represents is noted. Variables are coded so that a higher score on an item implies greater severity. IDS-SR, Inventory of Depressive Symptomatology Self-Report; SIAS, Social Interaction Anxiety Scale; HRSA, Hamilton Rating Scale for Anxiety; Dimensions, Psychiatric Dimensions Questionnaire.

a. View of myself is measured negatively, with higher scores depicting a more negative self-view based on self-blaming and criticism, and ruminating on personal shortcomings and defects.

b. Suicidal ideation is a broad concept measured as suicidal thoughts and intent. It ranges from mild infrequency of thoughts of death and suicide to more severe suicidal intent. Frequent thoughts of suicide and death are combined with making plans or attempting suicide.

### Statistical analyses

Analyses were performed in R version 3.6.1 for Windows (R Foundation for Statistical Computing, Vienna, Austria; see https://www.R-project.org/) .^23^ We modelled the longitudinal relationships between variables with a CLPN, a model designed by Rhemtulla et al,^22^ which combines network modelling with cross-lagged panel modelling. This allows individual items to affect other items over time, using two-wave panel data by measuring cross-lagged (i.e. the effect of a symptom at time point 1 on another symptom at time point 2) and autoregressive (i.e. the effect of a symptom at time point 1 on itself at time point 2) effects. Age at time point 1, gender, diagnostic category and treatment were included as covariates. Twenty-two participants had missing data, which was missing at random according to Little's Missing Completely at Random test (χ^2^ = 130.55, d.f. = 155, *P* = 0.92). CLPN modelling requires complete-case analysis, so missing data was imputed with the random forest imputation algorithm, implemented with the R package *missForest*.^24^

To estimate the CLPN, we computed autoregressive and cross-lagged coefficients with a series of regularised regressions, using the penalised maximum likelihood with a LASSO penalty.^25^ This results in a sparse network, which reduces overfitting and false positive edges by shrinking all edge weights and setting the smallest to zero. The network was estimated with the R package *glmnet.*^26^ After estimation, the network was visualised as a directed network with the R package *qgraph*.^27^ Arrows demonstrate the direction of temporal relationships: solid bluearrows represent positive relationships, dashed red arrows represent negative relationships and thicker lines represents stronger relationships between nodes. Placement of the nodes is determined by the Fruchterman–Reingold algorithm,^28^ in which nodes that are more connected are placed closer together.

Two measures of centrality were computed with the R package *bootnet*:^29^ cross-lagged out-expected influence (out-EI) and cross-lagged in-expected influence (in-EI). Out-EI is calculated as the sum of all outgoing edge strengths connected to a node, measuring how much a node influences other nodes. In-EI is calculated as the sum of all incoming edge strengths connected to a node, measuring how much a node is influenced by other others. Clinically, out-EI could be considered a treatment target, whereas in-EI could be considered an important treatment outcome.

Stability checks were conducted to assess the accuracy of edge weights, differences between edges and centralities, and the stability of centralities, using *bootnet* as detailed in Epskamp et al^29^ and with a custom function developed by Funkhouser et al.^5^

For sensitivity analyses, a control network without misophonia was estimated, given that it was the largest group (39.6% of the sample) and may affect the whole-sample estimates. Centralities were computed and stability checks were conducted for this control network. Similarities between the main and control network were evaluated, using the correlation between edge lists as a global measure of network similarity, the percentage of individual edges that are replicated, correlations of centralities between networks and replication of the most central symptoms.

## Results

### Sample characteristics

Data from 222 participants collected between 2012 and 2022 were included in the analyses. The distribution of the primary diagnosis reflects the naturalistic patient population of the Amsterdam UMC. Sample characteristics can be seen in Table 2. Symptom variables scores are shown in Supplementary Table 2. Furthermore, participants who completed both measurements were compared with participants who completed only the first measurement as a sensitivity analysis. Results can be viewed in Supplementary Table 4. There was a significant difference in age and in the distribution of diagnosis and medication. Except for feeling threatened or paranoid, there were no significant differences in symptom severity.

**Table 2:** Demographic and clinical characteristics of participants

Characteristics	Baseline
Age (years), mean (s.d.)	
Baseline	38.8 (15.4)
Follow-up	39.9 (15.4)
Months between measures, mean (s.d)	12.1 (2.4)
Gender, women, *n* (%)	121 (54.4)
Completed education^a^, *n* (%)	
Low	19 (8.6)
Middle	50 (22.5)
High	144 (64.9)
Unknown	9 (4.1)
DSM diagnostic category, *n* (%)	
Schizophrenia spectrum and other psychotic disorders	21 (9.5)
Depressive disorder	37 (16.8)
Anxiety disorder	9 (4.04)
Obsessive–compulsive and related disorders	41 (18.5)
Misophonia (impulse-control disorder not otherwise specified)	88 (39.6)
Bipolar disorder	14 (6.31)
Other disorders	12 (5.4)
Comorbidity, *n* (%)	54 (24.3)
Presence of treatment, *n* (%)	
None	12 (5.4)
Before time point 1	158 (71.2)
Between time points 1 and 2	52 (23.4)
Medication, *n* (%)	
Antidepressants	61 (27.8)
Antipsychotics	24 (10.8)
Benzodiazepines	3 (1.4)
Psychostimulants	4 (1.8)
Mood stabilisers	4 (1.8)
Other (non-psychotropic)^b^	44 (19.8)
None	82 (36.9)
Psychotherapy and treatments, *n* (%)^c^	
Evidence-based treatments	178 (80.2)
Supplementary or alternative interventions	161 (72.5)

a. Based on the Verhage coding of educational levels : low (1–4: less than or equal to primary education or low-level secondary education), middle (5: average-level secondary education) and high (6–7: high-level secondary education or university degree).

b. Other medication includes anti-inflammatory, antihistamine, anti-epilepsy, contraceptives, cholesterol medication, corticosteroids, dopamine agonists and various supplements.

c. Participants often followed multiple types of treatment. Evidence-based treatments include cognitive and behavioural therapies, trauma therapies, system therapy, schema therapy and psychotherapies. Supplementary or alternative interventions include talk therapy, counselling, coaching, expressive or creative therapies, skills trainings, psychodynamic therapy, reintegration support, peer support, ambulant care and lifestyle interventions.

### CLPN analysis

**Fig. 1:**
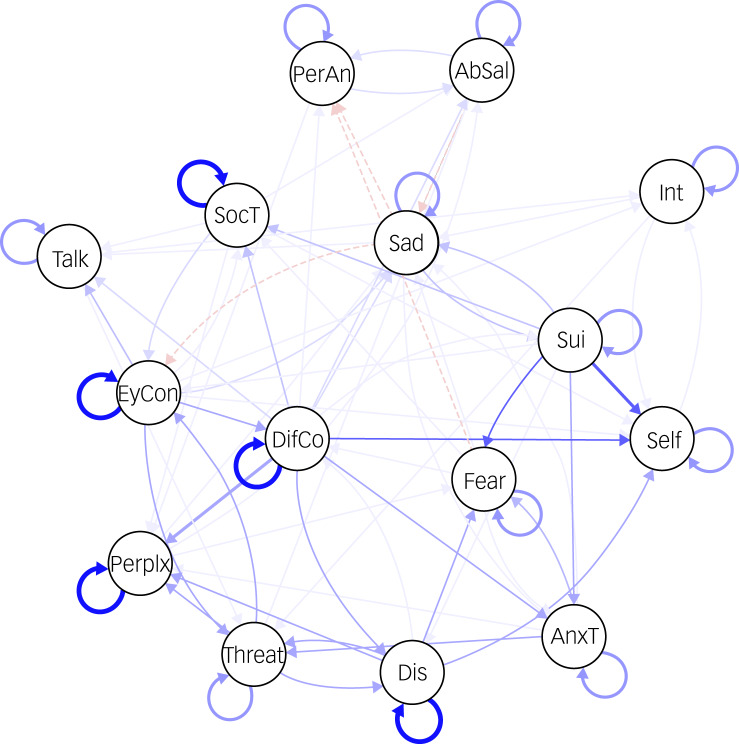
Transdiagnostic cross-lagged panel network of symptoms with autoregressive effects. Nodes represent the variables included in the network and edges with arrows indicate a directed association between nodes. Solid edges represent positive associations and dashed edges represent negative associations. AbSal, aberrant salience; AnxT, anxious tension; DifCo, difficulty mixing with co-workers; Talk, difficulty talking with others; Dis, difficulty disagreeing; EyCon, difficulty making eye contact; Int, interest; PerAn, perceptual anomalies; Perplx, perplexity, lack of natural evidence; Self, self-view; Sui, suicidal ideation; SocT, social tension; Threat, feeling threatened or paranoid.

The CLPN of psychopathological symptoms is visualised in Fig. 1, which presents cross-lagged and autoregressive relationships.

All nodes had at least one connection to another node, whether as predictor or outcome, resulting in 79 non-zero cross-lagged edges, of which 92.4% were positive. The strongest cross-lagged edge was between more baseline suicidal ideation predicting higher follow-up negative self-view (B = 0.36). The strongest autoregressive relationships pertained to social anxiety items: difficulty making eye contact (B = 0.57), difficulty disagreeing with others (B = 0.54) and social tension (B = 0.53). These were observed as the strongest edges in the matrix of the edge weights (i.e. regression coefficients), which can be seen in Supplementary Table 3. There are some other notable connections representing potential feedback loops between sadness and suicidal ideation, between difficulty disagreeing with others and difficulty mixing with co-workers, between feeling threatened (paranoia) and difficulty disagreeing with others, and between feeling threatened and difficulty making eye contact. These are expanded upon and discussed in Supplementary Appendix 2.

The effects of the covariates can be seen in Supplementary Table 3. Treatment had the most effect on symptoms in the network, whereas age, gender and diagnosis had few relationships with symptoms. Presence of treatment before time point 1 was related to increased severity of a few symptoms, mostly related to anxiety, whereas presence of treatment between time points 1 and 2 was related to decreased severity of a few symptoms, mostly related to depression. Neither treatment covariate had an effect on attenuated psychotic symptoms.

**Fig. 2:**
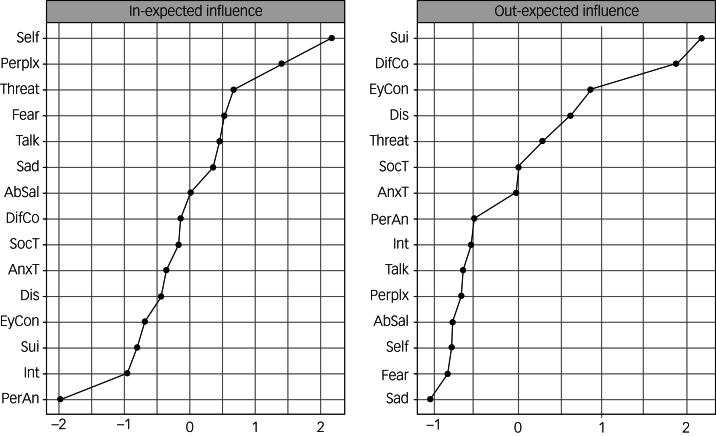
Cross-lagged centrality plots of out-expected influence and in-expected influence. The nodes are denoted on the *y*-axis and the standardised centrality coefficients are denoted on the *x*-axis. Higher *z*-scores indicate higher centrality. Because of the low stability, out-expected influence should not be interpreted. AbSal, aberrant salience; AnxT, anxious tension; DifCo, difficulty mixing with co-workers; Talk, difficulty talking with others; Dis, difficulty disagreeing; EyCon, difficulty making eye contact; Int, interest; PerAn, perceptual anomalies; Perplx, perplexity, lack of natural evidence; Self, self-view; Sui, suicidal ideation; SocT, social tension; Threat, feeling threatened or paranoid.

The centrality plots can be seen in Fig. 2. Stability was low for out-EI, but strong for in-EI (correlation-stability coefficient of 0.21 and 0.52, respectively). A correlation-stability coefficient should not be below 0.25, and should preferably be above 0.5.^29^ Therefore, out-EI is not interpreted. Negative self-view had the highest predictability and had significantly higher in-EI than ten out of 15 other symptoms (Supplementary Figure 3), suggesting that self-view tends to be influenced by other symptoms.

The control network without misophonia (*n* = 134) replicated the relationship between baseline suicidal ideation and follow-up self-view as the strongest edge (B = 0.33). The strongest autoregressive relationships of social tension (B = 0.64), difficulty making eye contact (B = 0.54) and difficulty disagreeing with others (B = 0.51) were also replicated. The edges of the main and control network were strongly correlated (*r* = 0.81): 90% of the edges in the control network were replicated in the main network and 64% of the edges in the main network were replicated in the control network. Negative self-view also had the highest predictability (in-EI), and correlation of overall out-EI was *r* = 0.77 and overall in-EI was *r* = 0.98 between the main and control networks. However, the correlation-stability coefficients for the control network are low, so results on centralities should not be interpreted (out-EI = 0.13, in-EI = 0.21). This is most likely because of the small sample size. The control network, centrality plots, and stability and difference tests can be viewed in Supplementary Figures 5–10.

## Discussion

This study aimed to investigate two-wave longitudinal relationships over an average of 12 months between individual symptoms, using a CLPN model in a transdiagnostic sample of individuals with psychiatric disorders. Interactions between symptoms from the different dimensions were also observed in the network, further supporting the co-occurrence of depression, (social) anxiety and attenuated psychotic symptoms. The strongest cross-lagged and autoregressive edges (suicidal ideation predicting negative self-view and self-reinforcing social anxiety symptoms) will be the focus of the discussion. Centrality analyses also detected self-view as a highly predictable node. These results were obtained by accounting for diagnosis as a control variable and were replicated in the control network without misophonia, which may potentially suggest that they are transdiagnostic. This supports the expectation that symptoms interact in ways that do not adhere to diagnostic categories.

An interesting relationship in the network was between more baseline suicidal ideation predicting higher follow-up negative self-view. As a note, suicidal ideation is measured in this study as a broad concept that ranges from thoughts of death to suicide attempts, and should be interpreted with caution as it does not clearly differentiate between ideation and actual attempts (see Table 1). Although research supports an association between suicidal ideation and self-esteem, longitudinal studies find that self-esteem predicts suicidal ideation,^30,31^ which is the opposite of what we found. A potential explanation for our findings is that individuals may feel shame or embarrassment for contemplating or attempting suicide,^32,33^ also known as self-stigma, which is associated with lower self-esteem.^34^ Additionally, suicidal ideation is often associated with feelings of burdensomeness,^35^ which are related to low self-esteem and self-hate.^36^ Negative self-view may possibly be a reflection of self-stigma and perceived burdensomeness as a result of suicidal ideation. Focusing on self-compassion as an intervention for dealing with suicidality may be worthwhile because it can potentially influence self-esteem and other related factors, such as self-stigma and perceived burdensomeness.^37,38^ Because of the conflation of within- and between-participant effects prevalent in CLPN models, cross-lagged edges should be interpreted with caution.

Other strong edges pertained to social anxiety symptoms, especially difficulty making eye contact, difficulty disagreeing with others and social tension, which had the strongest autoregressive effects. This suggests they are the most self-reinforcing symptoms in the network. Models of social anxiety point to a self-perpetuating cycle in interpersonal situations, such that an individual might behave in anticipation of or according to their expectations of how another individual might react or behave.^39,40^ Cognitive biases and using safety behaviours, such as avoiding eye contact or seeking approval, reinforce and maintain social anxiety.^41^ Furthermore, the strength of these symptoms could indicate their relevance in a transdiagnostic manner. For instance, social anxiety is prevalent in individuals with other disorders, such as psychosis,^42^ bipolar disorder^43^ and depression.^44^ These could reflect more general difficulties with social interaction.^45^

Self-view was a main transdiagnostic outcome in this network, as suggested by its high predictability. This means that transdiagnostically, many other symptoms influenced self-view, and participants had a lower self-view when they experienced these other symptoms. Although more attention is given to self-esteem as a risk factor or development mechanism for psychopathology, self-esteem may also be an outcome of psychopathology.^46,47^ The current finding is in line with the scar model, which posits that psychopathology tends to deplete psychological resources, leaving scars that distort an individual's self-concept. Having a psychiatric disorder could potentially lower self-esteem.^48^ The vulnerability model, in which self-esteem predicts psychopathology, has more support,^49^ but our results do not align with this. However, the scar and vulnerability models are not mutually exclusive, and a bidirectional relationship is possible.^47^

In our network, self-view was mostly predicted by nodes related to depression and social anxiety symptoms. Considering that depression and social anxiety symptoms can affect many areas of life, such as psychosocial functioning,^50,51^ a negative self-view might not be a direct outcome of these factors. Instead, it might be a reflection of the negative consequences of living with a psychiatric disorder. Individuals with psychiatric disorders often have limited access to work, education and social activities; activities which often are considered meaningful. For instance, employment is associated with higher self-esteem.^52^ Not being able to participate in society could, therefore, lead to loss of self-esteem. Living with psychiatric disorders can also lead to demoralisation, as accepting the realities of mental illness and its consequences can affect self-esteem.^53^ This is especially so if one internalises that mental illness means inadequacy, incompetence and that there is something inherently wrong with oneself.^54^ Emphasising psychosocial rehabilitation and recovery-oriented care concepts such as empowerment, hope and inclusion in recovery may lead to better long-term outcomes, including improvements in self-esteem.^55^

Of the covariates, treatment was mostly strongly related with symptoms. Treatment before the baseline measurement was related to higher sadness and (social) anxiety symptoms. A potential reason for this may be increased self-reflection and insight, which can have a paradoxical effect of increasing symptoms, perhaps through self-stigma.^56^ The AMC is also a tertiary care institution, and tends to treat individuals with more severe cases who typically have a previous history of treatment. Treatment between the baseline and follow-up measurements was related to lower symptom severity, especially negative self-view, lack of interest and suicidal ideation. Considering the transdiagnostic nature, this may be related to general improvements in well-being and quality of life. Factors such as duration and type of treatment were not included in the treatment covariates, so these findings must be interpreted cautiously.

### Strengths and limitations

A main strength of this study is that it employs a transdiagnostic approach that cuts across multiple diagnostic spectra and focuses on individual symptoms rather than on sum scores or diagnoses. Broad categories, whether sum scores or diagnoses, can lead to a loss of information on how specific symptoms or mental states, irrespective of diagnosis, interact with each other. Furthermore, the analyses were controlled for diagnoses and were repeated in a diagnostic subsample without misophonia to check for the potential influence of misophonia, which is the largest group. However, future research should conduct analyses of individual diagnostic categories to determine a true transdiagnostic nature. Finally, conducting a CLPN model is a step forward from cross-sectional partial correlation networks, as it allows for directed relationships. Longitudinal investigations are necessary to investigate the dynamic nature of psychopathology, and determining temporal order is one step toward determining causality.

The results of this paper should be interpreted with the following limitations in mind. First, CLPNs can be influenced by limitations pertaining to traditional cross-lagged panel models and network models, as explained by Rhemtulla et al.^22^ A main limitation is that CLPN models conflate within- and between-participant effects, which can bias results if variables contain stable individual differences. This means that cross-lagged effects may be produced among correlated variables that have no causal relationships. Methods that can separate these effects require at least three waves of data and require more research, but include mean-centring data across time points or fitting a latent factor to repeated observations in a random-intercept, cross-lagged panel model. Furthermore, estimates can be affected by sampling frequency, so relationships in this network should be interpreted in light of the 1-year time lag represented in this study. Moreover, two time points do not allow for analysing dynamic bidirectional relationships. Future research therefore could include intensive time-series designs measuring hourly or daily fluctuations, and multi-wave longitudinal studies to measure longer-term changes to account for differences in frequency of change. Furthermore, the small sample size is a limitation, which did not allow us to compare individual networks per diagnostic category. However, diagnosis was included as a covariate in the network to account for potential differences. The small sample size may have also affected the stability estimates and should therefore be interpreted cautiously.

Another potential limitation is selection bias. Participants who completed both measurements may differ from participants who completed only the first measurement, especially as completing the follow-up measurement was not necessary for participation in the study. For instance, we found a different in age, diagnosis, medication and feeling threatened or paranoid. This further contributed to the small sample size. Furthermore, misophonia comprised a large percentage of the sample, which may also be a selection bias. However, the control network without the misophonia subsample demonstrated a moderate correlation with the main network and substantially replicated the main findings. Participants could also participate at any point of their treatment trajectory, which could affect stationarity, but we controlled for this in the network. This reflects the naturalistic nature of the study, which can more closely reflect clinical reality, but also introduce more variability in variables, such as age, treatment and diagnostic category. Finally, most of the nodes contained only one item, which might not be sufficient to capture some of the more complex concepts, including suicidal ideation.

To conclude, this study gives insight into two-wave longitudinal interactions between individual symptoms, which cut across diagnostic categories. Overall, all nodes in the network both predicted and were influenced by at least one other node, which resulted in numerous unique associations. This demonstrates the dynamic complexity of psychopathology, and further supports the importance of investigating symptom interactions of different psychopathological dimensions over time and across disorders. Potential mechanisms of these relationships need to be elucidated, and models should be extended to include measures of psychosocial functioning and daily life, to investigate how these are affected by symptoms.

## Data Availability

The data that support the findings of this study are available on request from the Across Consortium Scientific Coordinator at across@amsterdamumc.nl. The data requests will be discussed in the Across Executive board. The data are not publicly available due to the clinical and confidential nature of the data.

## Supplementary material

For supplementary material accompanying this paper visit http://doi.org/10.1192/bjo.2022.516. click here to view supplementary material
