# Supplementary material for: The interplay between psychopathological symptoms: transdiagnostic cross-lagged panel network model
Source: BJPsych Open. 2022 Jun 27;8(4):e116. doi: 10.1192/bjo.2022.516 (PMC9301766; doi:10.1192/bjo.2022.516)
Supplement: Supplementary file 1 [file S2056472422005166sup001.docx]

Supplementary Materials

**The interplay between psychopathological symptoms: A transdiagnostic cross-lagged panel network model**

UnYoung Chavez-Baldini, Karin J.H. Verweij, Derek de Beurs, Claudi L.H. Bockting, Anja Lok, Arjen L. Sutterland, Geeske van Rooijen, Guido van Wingen, Across Consortium, Damiaan Denys, Nienke C. Vulink, Dorien H. Nieman

Contents

[**Appendix 1. Item Selection Procedure** 2](#_Toc101864525)

[**Appendix 2. Feedback loops** 3](#_Toc101864526)

[**Table S1**. Specific diagnoses per diagnostic category 5](#_Toc101864527)

[**Table S2.** Symptom scores: Mean (SD), range 6](#_Toc101864528)

[**Table S3.** Edge weights matrix 8](#_Toc101864529)

[**Table S4**. Comparison between participants with and without follow-up measure 9](#_Toc101864530)

[**Figure S1**. Accuracy of the estimated edges 10](#_Toc101864531)

[**Figure S2.** Difference tests of edges 11](#_Toc101864532)

[**Figure S3.** Difference tests of centralities 12](#_Toc101864533)

[**Figure S4.** Stability of the centrality measures 13](#_Toc101864534)

[**Figure S5**. Control cross-lagged network without misophonia with autoregressive effects 14](#_Toc101864535)

[**Figure S6**. Cross-lagged centrality plots for control network without misophonia. 15](#_Toc101864536)

[**Figure S7**. Accuracy of the estimated edges for control network without misophonia 16](#_Toc101864537)

[**Figure S8.** Difference tests of edges for control network without misophonia 17](#_Toc101864538)

[**Figure S9.** Difference tests of centralities for control network without misophonia 18](#_Toc101864539)

[**Figure S10.** Stability of the centrality measures for control network without misophonia 19](#_Toc101864540)

# **Appendix 1. Item Selection Procedure**

Following Rhemtulla, van Bork (1)’s recommendation, an item selection procedure was performed, in which the number of items was reduced. This is done to make the network results more interpretable as many items may have conceptual overlap. This item selection was done before any analyses were conducted and was based on a combination of content-based and data-driven reduction. If available, we first used short-form versions of questionnaires to guide item selection: QIDS-SR_16_ (2), SIAS-6 (3), and HAM-A_6_ (4). This reduced the total number of items from all questionnaires combined from 79 to 32. Next, we selected items that pertained to psychological symptoms, meaning we removed items pertaining to somatic or physical symptoms, such as sleep. This further reduced the number of items to 18. Next, we performed a node redundancy procedure using a weighted topological overlap approach and adaptive alpha multiple comparisons correction to determine which items are redundant. This procedure was conducted with the node.redundant function available in the *EGAnet* package (5). Item wording and theory was also taken into consideration when determining which nodes to combine as was suggested in Rhemtulla, van Bork (1). This further reduced the 18 items to 15 items. The final list of items can be viewed in Table 1 in the main manuscript.

# **Appendix 2. Feedback loops**

**Results**

A couple of potential feedback loops were present in the current network. More sadness at baseline predicted more suicidal ideation at follow-up (B= 0.10) and more suicidal ideation at baseline predicted more sadness at follow-up (B= 0.14). Feeling more threatened at baseline predicted more difficulty disagreeing with others at follow-up (B= 0.08) and more difficulty disagreeing with others at baseline predicted feeling more threated at follow-up (B= 0.12). Feeling more threatened at baseline also predicted more difficulty making eye contact at follow-up (B= 0.08) and more difficulty making eye contact at baseline predicted feeling more threated at follow-up (B= 0.10). More difficulty disagreeing with others at baseline predicted more difficulty mixing with co-workers (B= 0.07) and more difficulty mixing with co-workers predicted more difficulty disagreeing with others (B= 0.12). Lastly, more perceptual anomalies predicted more aberrant salience (B= 0.11) and more aberrant salience predicted more perceptual anomalies (B= 0.07). These could suggest tentative feedback loops as explained in Kuipers, Moffa (6). If variable A at baseline affects variable B at follow-up, and B at baseline affects A at follow-up, we could potentially infer a feedback loop. Disentangling the effect of synchronous correlation (^r^A1B1 ^r^A2B2) from causal cross-item influence is difficult, however, so this should be interpreted cautiously. Furthermore, at least three waves are necessary to detect true feedback loops.

**Discussion**

A couple of potential transdiagnostic feedback loops were observed in the network. One positive feedback loop was between sadness and suicidal ideation. Sadness and negative affect have been shown to related to and predict suicidal thoughts (7-9). Unfortunately, there is a dearth of research investigating suicidal ideation as a predictor as it is often considered an outcome. Moreno-Küstner, Jones (10) found that suicidal ideation predicted persistent depression, and considering that sadness is a core symptom of depression, it may suggest that suicidal ideation could predict sadness. However, more bidirectional research is necessary between negative affect and suicidal ideation.

Two other positive feedback loops were between feeling threatened and difficulty disagreeing with others between feeling threatened and difficulty making eye contact. A link between social anxiety and exceptional or psychotic-like experiences related to anticipating harm from others, paranoia, and persecutory delusions has been demonstrated in previous research (11-13), which support these two feedback loops found in the network. A fear of others characterized by social threat is an underlying common factor for those with social anxiety and paranoia or persecutory delusions (13) and may explain these relationships.

The potential feedback loop between difficulty disagreeing with others and difficulty mixing with co-workers could be explained by an overarching relationship between social anxiety and self-protective strategies, including reduce self-disclosure and less assertiveness (14-17). Mingling with others, including co-workers, can involve self-disclosure, and expressing disagreement is not only a form of disclosing one’s opinions, it also requires asserting oneself. As such, these self-protective strategies could lead to feedback loops of negative behaviors that perpetuate each other.

Lastly, aberrant salience and perceptual anomalies has a well-established link in the literature. Models of psychosis posit that aberrant salience can lead to perceptual biases and psychotic-like experiences (e.g., 18, 19) and are especially related to positive psychotic symptoms and experiences (20). While there is support for aberrant salience leading to perceptual anomalies and generally positive psychotic-like experiences, there is a lack of research on the converse relationship. Given that aberrant salience refers to giving excessive attention or importance to neutral or irrelevant stimuli, perceptual anomalies, or perceiving what others cannot perceive, could increase aberrant salience by making that perception more important in that individual’s mind.

While these relationships only indicate potential feedback loops rather than reflect true feedback loops, these findings further point to the dynamic nature of psychopathology.

# **Table S1**. Specific diagnoses per diagnostic category

| **Diagnosis** | **n** |
| --- | --- |
| **Schizophrenia spectrum and other psychotic disorders (n=21)** | |
| Schizophrenia, Paranoid Type | 7 |
| Schizophrenia or Psychotic Disorder NOS | 10 |
| Schizophreniform Disorder | 1 |
| Schizoaffective Disorder | 2 |
| Delusional Disorder | 1 |
| **Depressive disorders (n=37)** | |
| Major Depressive Disorder, Recurrent | 19 |
| Major Depressive Disorder, Single Episode | 9 |
| Dysthymic Disorder | 3 |
| Cyclothymic Disorder | 1 |
| Depressive Disorder NOS | 5 |
| **Anxiety disorders (n=9)** | |
| Generalized Anxiety Disorder | 3 |
| Panic Disorder | 1 |
| Posttraumatic Stress Disorder | 4 |
| Anxiety Disorder NOS | 1 |
| **Obsessive-compulsive and related disorders (n=41)** | |
| Body Dysmorphic Disorder | 3 |
| Skin Picking (Excoriation Disorder) | 1 |
| Trichotillomania | 4 |
| Obsessive-Compulsive Disorder | 33 |
| **Misophonia (Impulse control disorder NOS)** | |
| Misophonia | 88 |
| **Bipolar disorders (n=14)** | |
| Bipolar I Disorder | 6 |
| Bipolar II Disorder | 7 |
| Bipolar Disorder NOS | 1 |
| **Other disorders (n=12)** | |
| Substance use disorders | 3 |
| Neurodevelopmental disorders | 5 |
| Impulse control disorders | 3 |
| Adjustment disorder | 1 |

Note: Misophonia is not an official DSM diagnosis.

# **Table S2.** Symptom scores: Mean (SD), range

| **Measure** | **All participants (N=222)** | **Schizophrenia/psychotic disorders**  **(n= 21)** | **Depressive disorders**  **(n= 37)** | **Anxiety disorders**  **(n= 9)** | **OCD**  **(n= 41)** | **Misophonia**  **(n= 88)** | **Bipolar**  **(n= 14)** | **Other**  **(n= 12)** | **Missing (%)** |
| --- | --- | --- | --- | --- | --- | --- | --- | --- | --- |
| **Baseline** | | | | | | | | | |
| Sad | 1.05 (0.9), 0-3 | 0.81 (0.87), 0-3 | 1.97 (0.93), 0-3 | 1 (0.5), 0-2 | 1.12 (0.95), 0-3 | 0.73 (0.7), 0-3 | 0.5 (0.65), 0-2 | 1.42 (1.16), 0-3 | 13.33 |
| Self-view | 1.12 (1.2), 0-3 | 0.86 (1.28), 0-3 | 1.97 (1.09), 0-3 | 1 (1), 0-3 | 1.39 (1.28), 0-3 | 0.66 (1.03), 0-3 | 1 (1.24), 0-3 | 1.58 (1.38), 0-3 | 13.33 |
| Suicidal ideation | 0.53 (0.8), 0-3 | 0.24 (0.54), 0-2 | 1.38 (1.01), 0-3 | 0.44 (0.73), 0-2 | 0.44 (0.74), 0-3 | 0.23 (0.55), 0-2 | 0.57 (0.94), 0-3 | 0.83 (1.11), 0-3 | 13.33 |
| Interest | 0.69 (0.9), 0-3 | 0.71 (0.78), 0-3 | 1.65 (1.11), 0-3 | 1.11 (0.93), 0-3 | 0.57 (0.8), 0-3 | 0.24 (0.47), 0-2 | 0.86 (0.86), 0-3 | 1 (1.04), 0-3 | 13.33 |
| Difficulty making eye contact | 0.8 (1.1), 0-4 | 1.05 (1.32), 0-4 | 1.3 (1.41), 0-4 | 0.78 (1.3), 0-3 | 0.76 (0.97), 0-4 | 0.58 (0.96), 0-3 | 0.64 (1.08), 0-3 | 0.83 (0.94), 0-3 | 13.33 |
| Difficulty mixing with co-workers | 0.88 (1), 0-4 | 1.05 (1.24), 0-3 | 1.62 (1.23), 0-4 | 0.78 (1.2), 0-3 | 0.76 (0.89), 0-3 | 0.58 (0.8), 0-3 | 0.86 (0.86), 0-2 | 1 (0.95), 0-3 | 13.33 |
| Social tension | 1.14 (1.2), 0-4 | 0.71 (0.87), 0-3.5 | 1.5 (0.98), 0-4 | 0.56 (0.77), 0-2 | 0.76 (0.91), 0-3 | 0.54 (0.71), 0-3 | 0.64 (0.72), 0-2 | 0.88 (0.86), 0-2.5 | 13.33 |
| Difficulty talking with others | 0.78 (0.9), 0-4 | 0.76 (0.94), 0-4 | 1.68 (1.18), 0-4 | 0.56 (1.13), 0-3 | 0.66 (0.76), 0-2 | 0.8 (0.87), 0-3 | 0.79 (0.89), 0-3 | 0.83 (1.27), 0-4 | 13.33 |
| Difficulty disagreeing | 0.91 (1), 0-4 | 0.67 (1.11), 0-4 | 2.03 (1.26), 0-4 | 0.89 (1.36), 0-4 | 1.22 (1.15), 0-4 | 0.93 (1.07), 0-4 | 1 (0.88), 0-3 | 0.92 (1.08), 0-3 | 13.33 |
| Anxious tension | 1.19 (1.2), 0-4 | 0.81 (1.21), 0-4 | 1.96 (1.44), 0-4 | 2.33 (0.94), 1-3.5 | 1.35 (1.16), 0-4 | 0.83 (0.94), 0-3.5 | 0.93 (1.22), 0-4 | 1.04 (1.16), 0-3 | 16.67 |
| Fear | 0.89 (1.2), 0-4 | 0.43 (0.75), 0-3 | 1.51 (1.48), 0-4 | 1.44 (1.42), 0-4 | 1.24 (1.2), 0-4 | 0.61 (1.02), 0-4 | 0.79 (1.19), 0-4 | 0.42 (0.51), 0-1 | 16.67 |
| Perceptual anomalies | 1.42 (1.9), 0-8 | 1.85 (1.77), 0-7 | 0.93 (1.65), 0-6 | 2.72 (2.51), 0-6 | 0.87 (1.73), 0-8 | 1.83 (2.15), 0-8 | 0.57 (1.16), 0-4 | 1.04 (1.39), 0-4 | 43.33 |
| Perplexity | 2.05 (2), 0-8 | 2.18 (2.11), 0-7 | 2.92 (2.32), 0-8 | 2.78 (2.83), 0-7 | 2.25 (1.92), 0-8 | 1.43 (1.48), 0-6 | 2.14 (2.11), 0-7.5 | 2.36 (1.67), 0-6.5 | 43.33 |
| Aberrant salience | 1.48 (1.9), 0-8 | 2.12 (2.25), 0-8 | 1.18 (1.42), 0-5 | 2.49 (2.83), 0-7 | 1.24 (2.03), 0-8 | 1.42 (1.62), 0-8 | 1.5 (2.24), 0-8 | 1.63 (1.98), 0-6 | 43.33 |
| Threatened; paranoia | 0.83 (1.4), 0-8 | 1.51 (2.25), 0-8 | 1.13 (1.76), 0-7 | 0.52 (0.71), 0-2 | 1.04 (1.55), 0-8 | 0.52 (0.74), 0-3 | 0.29 (0.73), 0-2 | 1.12 (1.91), 0-6 | 43.33 |

**Table S2.** Symptom scores: Mean (SD), continued

| **Measure** | **All participants (N=222)** | **Schizophrenia/psychotic disorders**  **(n= 21)** | **Depressive disorders**  **(n= 37)** | **Anxiety disorders**  **(n= 9)** | **OCD**  **(n= 41)** | **Misophonia**  **(n= 88)** | **Bipolar**  **(n= 14)** | **Other**  **(n= 12)** | **Missing (%)** |
| --- | --- | --- | --- | --- | --- | --- | --- | --- | --- |
| **Follow-up** | | | | | | | | | |
| Sad | 0.8 (0.8), 0-3 | 0.81 (0.81), 0-3 | 1.23 (0.91), 0-3 | 0.44 (0.53), 0-1 | 0.81 (0.84), 0-3 | 0.62 (0.71), 0-3 | 1 (1.04), 0-3 | 0.75 (0.62), 0-2 | 20.00 |
| Self-view | 0.86 (1.2), 0-3 | 0.48 (0.93), 0-3 | 1.92 (1.25), 0-3 | 0.33 (0.5), 0-1 | 0.85 (1.21), 0-3 | 0.43 (0.88), 0-3 | 1.29 (1.38), 0-3 | 1.42 (1.31), 0-3 | 20.00 |
| Suicidal ideation | 0.38 (0.7), 0-3 | 0.43 (0.81), 0-2 | 0.86 (0.88), 0-3 | 0.56 (0.88), 0-2 | 0.25 (0.66), 0-3 | 0.17 (0.45), 0-2 | 0.64 (0.84), 0-2 | 0.42 (0.79), 0-2 | 20.00 |
| Interest | 0.48 (0.8), 0-3 | 0.67 (1.02), 0-3 | 0.86 (0.82), 0-3 | 0.22 (0.44), 0-1 | 0.47 (0.67), 0-2 | 0.22 (0.51), 0-3 | 0.71 (0.91), 0-3 | 0.83 (1.11), 0-3 | 20.00 |
| Difficulty making eye contact | 0.69 (1.1), 0-4 | 0.9 (1.22), 0-4 | 0.99 (1.24), 0-4 | 0.44 (1.01), 0-3 | 0.61 (0.95), 0-4 | 0.61 (1.05), 0-4 | 0.57 (1.09), 0-3 | 0.58 (0.67), 0-2 | 16.67 |
| Difficulty mixing with co-workers | 0.7 (1), 0-4 | 0.86 (1.15), 0-3 | 1.16 (1.19), 0-4 | 0.56 (1.33), 0-4 | 0.56 (0.81), 0-3 | 0.53 (1), 0-4 | 0.79 (0.7), 0-2 | 0.67 (0.78), 0-2 |  |
| Social tension | 0.72 (0.9), 0-4 | 0.64 (0.91), 0-3.5 | 1.36 (1.05), 0-3 | 0.39 (0.82), 0-2.5 | 0.68 (0.82), 0-3 | 0.56 (0.77), 0-4 | 0.68 (0.64), 0-2 | 0.54 (0.78), 0-2.5 | 16.67 |
| Difficulty talking with others | 0.81 (0.9), 0-4 | 0.95 (0.97), 0-3 | 1.32 (1.01), 0-3 | 0.56 (1.13), 0-3 | 0.61 (0.67), 0-2 | 0.69 (0.89), 0-4 | 0.71 (0.83), 0-3 | 0.92 (0.79), 0-2 | 16.67 |
| Difficulty disagreeing | 1.04 (1.2), 0-4 | 0.57 (0.87), 0-3 | 1.79 (1.3), 0-4 | 0.44 (0.88), 0-2 | 1.32 (1.25), 0-4 | 0.86 (1.09), 0-4 | 0.79 (0.7), 0-2 | 0.67 (0.78), 0-2 | 16.67 |
| Anxious tension | 0.89 (1.1), 0-4 | 0.43 (0.84), 0-2.5 | 1.35 (1.22), 0-4 | 1.33 (1.32), 0-4 | 1.01 (1.18), 0-4 | 0.71 (0.95), 0-4 | 0.86 (1.23), 0-3.5 | 0.79 (0.89), 0-2.5 | 16.67 |
| Fear | 0.65 (1), 0-4 | 0.29 (0.56), 0-2 | 1.25 (1.34), 0-4 | 0.78 (1.39), 0-4 | 0.85 (1.13), 0-4 | 0.41 (0.71), 0-4 | 0.71 (1.14), 0-3 | 0.33 (0.49), 0-1 | 16.67 |
| Perceptual anomalies | 1.16 (1.8), 0-8 | 1.38 (2.13), 0-8 | 0.72 (1.3), 0-6 | 1.56 (2.6), 0-6 | 0.78 (1.6), 0-8 | 1.52 (1.95), 0-7 | 0.93 (1.64), 0-6 | 0.83 (1.53), 0-5 | 16.67 |
| Perplexity | 1.76 (1.8), 0-7.5 | 1.62 (1.77), 0-7 | 2.44 (1.85), 0-7 | 1.83 (2.32), 0-6 | 1.71 (1.78), 0-7.5 | 1.36 (1.37), 0-7 | 2.54 (2.52), 0-7 | 2.12 (1.76), 0-5 | 16.67 |
| Aberrant salience | 1.39 (1.9), 0-8 | 1.57 (1.99), 0-6 | 1.36 (1.82), 0-7 | 2 (2.5), 0-6 | 1.15 (1.9), 0-8 | 1.55 (2.03), 0-8 | 1.14 (1.92), 0-7 | 0.67 (0.98), 0-3 | 16.67 |
| Threatened; paranoia | 0.81 (1.3), 0-8 | 0.9 (1.22), 0-4 | 1.13 (1.51), 0-7 | 1 (2.65), 0-8 | 0.83 (1.24), 0-6 | 0.53 (0.82), 0-4 | 0.86 (0.86), 0-2 | 1.5 (1.83), 0-5 | 16.67 |

*Note*: The Psychiatric Dimensions Questionnaire was introduced into the study at a later point, which explains the higher rates of missing data in the baseline scores of the attenuated psychotic symptoms.

# **Table S3.** Edge weights matrix

|  | **Sad** | **Self** | **Sui** | **Int** | **EyCon** | **DifCo** | **SocT** | **Talk** | **Dis** | **AnxT** | **Fear** | **PerAn** | **Perplx** | **AbSal** | **Threat** |
| --- | --- | --- | --- | --- | --- | --- | --- | --- | --- | --- | --- | --- | --- | --- | --- |
| **Sad** | .242 | .010 | .096 | .000 | -.097 | .000 | .000 | .064 | .000 | .034 | .000 | -.140 | .000 | .000 | .000 |
| **Self** | .000 | .209 | .000 | .037 | .000 | .000 | .000 | .000 | .004 | .000 | .000 | .000 | .000 | .000 | .000 |
| **Sui** | .136 | .358 | .287 | .000 | .062 | .017 | .110 | .000 | .037 | .089 | .108 | .000 | .000 | .000 | .000 |
| **Int** | .014 | .066 | .000 | .269 | .000 | .000 | .000 | .000 | .000 | .009 | .000 | .000 | .000 | .000 | .018 |
| **EyCon** | .056 | .029 | .000 | .035 | .566 | .142 | .011 | .145 | .000 | .000 | .004 | .000 | .000 | .003 | .101 |
| **DifCo** | .079 | .077 | .002 | .000 | .000 | .422 | .099 | .085 | .118 | .024 | .000 | .055 | .194 | .086 | .009 |
| **SocT** | .000 | .051 | .000 | .000 | .091 | .000 | .528 | .069 | .000 | .000 | .000 | .000 | .054 | .012 | .000 |
| **Talk** | .000 | .000 | .000 | .010 | .000 | .000 | .000 | .234 | .000 | .000 | .000 | .000 | .003 | .000 | .066 |
| **Dis** | .000 | .104 | .000 | .000 | .000 | .071 | .000 | .000 | .542 | .000 | .087 | .000 | .077 | .000 | .118 |
| **AnxT** | .017 | .000 | .000 | .000 | .000 | .000 | .005 | .000 | -.057 | .332 | .161 | .000 | .048 | .000 | .092 |
| **Fear** | .046 | .007 | .012 | .000 | .000 | .014 | .012 | .000 | .000 | .045 | .233 | -.110 | .000 | .000 | .000 |
| **PerAn** | .000 | .000 | .000 | .000 | .000 | .000 | .000 | .000 | .000 | .000 | .000 | .306 | .012 | .107 | .000 |
| **Perplx** | .021 | .000 | .000 | .000 | .000 | .000 | .000 | .000 | .000 | .000 | .018 | .000 | .394 | .035 | .000 |
| **AbSal** | -.027 | .000 | .000 | .000 | .000 | .000 | .000 | .000 | .000 | .000 | .000 | .074 | -.003 | .347 | .000 |
| **Threat** | .000 | .000 | .000 | .000 | .078 | .000 | .000 | .000 | .083 | .000 | .000 | .000 | .166 | .031 | .301 |
| **Age** | .000 | .000 | .000 | .000 | -.004 | .000 | .000 | .000 | .000 | .000 | .000 | .000 | .011 | .000 | .001 |
| **Gender** | .035 | .003 | .000 | .000 | .000 | .058 | .020 | .000 | .180 | .000 | .000 | .000 | .000 | .000 | .000 |
| **Diagnosis** | .000 | .000 | .000 | .000 | .000 | .000 | .000 | .000 | .000 | .000 | .000 | .000 | .056 | .000 | .000 |
| **Treat1** | .056 | .000 | .000 | .000 | .000 | .061 | .000 | .000 | .173 | .000 | .047 | .000 | .000 | .000 | .000 |
| **Treat2** | .000 | -.112 | -.107 | -.103 | .000 | .000 | .000 | -.006 | .000 | .000 | .000 | .000 | .000 | .000 | .000 |

Note: Independent variables are in rows and dependent variables are in columns. Autoregressive edges are presented along the diagonal. Covariates are presented in the last five rows.
Abbreviations: AbSal = aberrant salience, AnxT = anxious tension, DifCo = difficulty mixing with co-workers, Talk = difficulty talking with others, Dis = difficulty disagreeing, EyCon = difficulty making eye contact, Fear = fears, Int = interest, PerAn = perceptual anomalies, Perplx = perplexity; lack of natural evidence, Sad = sad, Self = self-view, Sui = suicidal ideation, SocT = social tension, Threat = feeling threatened; paranoia.

# **Table S4**. Comparison between participants with and without follow-up measure

| **Variable (Baseline)** | **Test statistic** |
| --- | --- |
| Age | t(322.43)= -4.84*** |
| Gender | χ²(1)= 0.01 |
| Diagnosis | χ²(6)= 26.09*** |
| Comorbidity | χ² (1)= 1.61 |
| Medication | χ²(5)= 13.28* |
| Sad | t(353.40)= 0.18 |
| Self | t(350.51)= 0.30 |
| Sui | t(341.82)= -0.87 |
| Int | t(361.57)= 1.04 |
| EyCon | t(361.28)= 1.32 |
| DifCo | t(378.27)= 0.75 |
| SocT | t(383.93)= 1.43 |
| Talk | t(365.62)= -0.14 |
| Dis | t(351.89)= -1.20 |
| AnxT | t(358.53)= 0.46 |
| Fear | t(377.59)= 1.40 |
| PerAn | t(378.91)= 0.55 |
| Perplx | t(361.70)= 0.59 |
| AbSal | t(390.05)= 1.60 |
| Threat | t(400.67)= 2.28* |

*Note*: Significance codes are * P≤ 0.05, **P ≤ 0.01, ***P ≤ 0.001

Participations without a follow-up measure (group 1) were younger than those with a follow-up measure (group 2). Differences in the distribution of the diagnostic category were minimal except that group 1 also had notably more patients with schizophrenia spectrum and other psychotic disorders. Group 1 also had a higher use of anti-depressants and benzodiazepines.


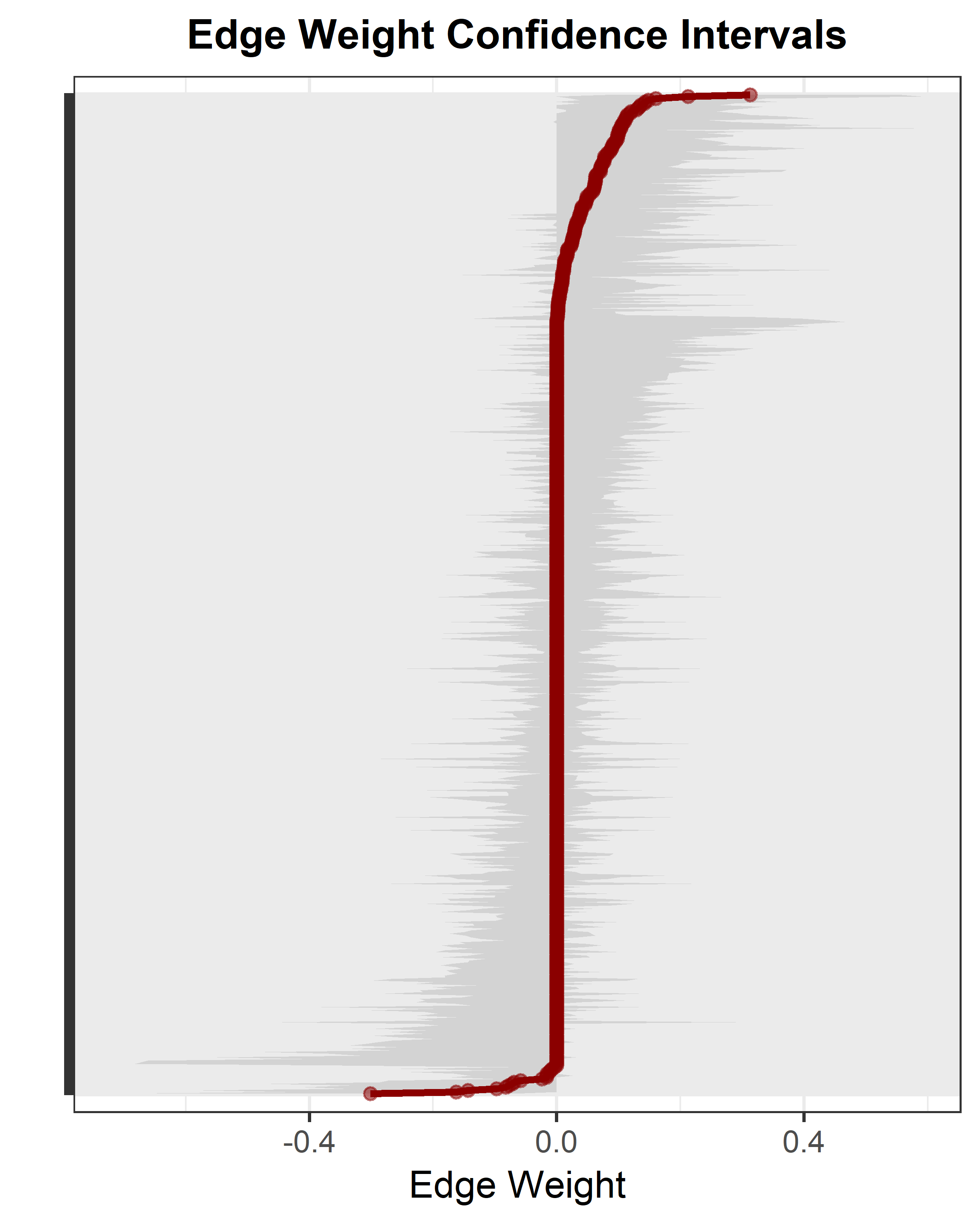


# **Figure S1**. Accuracy of the estimated edges

The x-axis shows the strength of the edge. The edges from the original network are shown in red and are arranged from most negative to most positive along the y-axis. The grey area represents confidence intervals based on the bootstrapped networks.


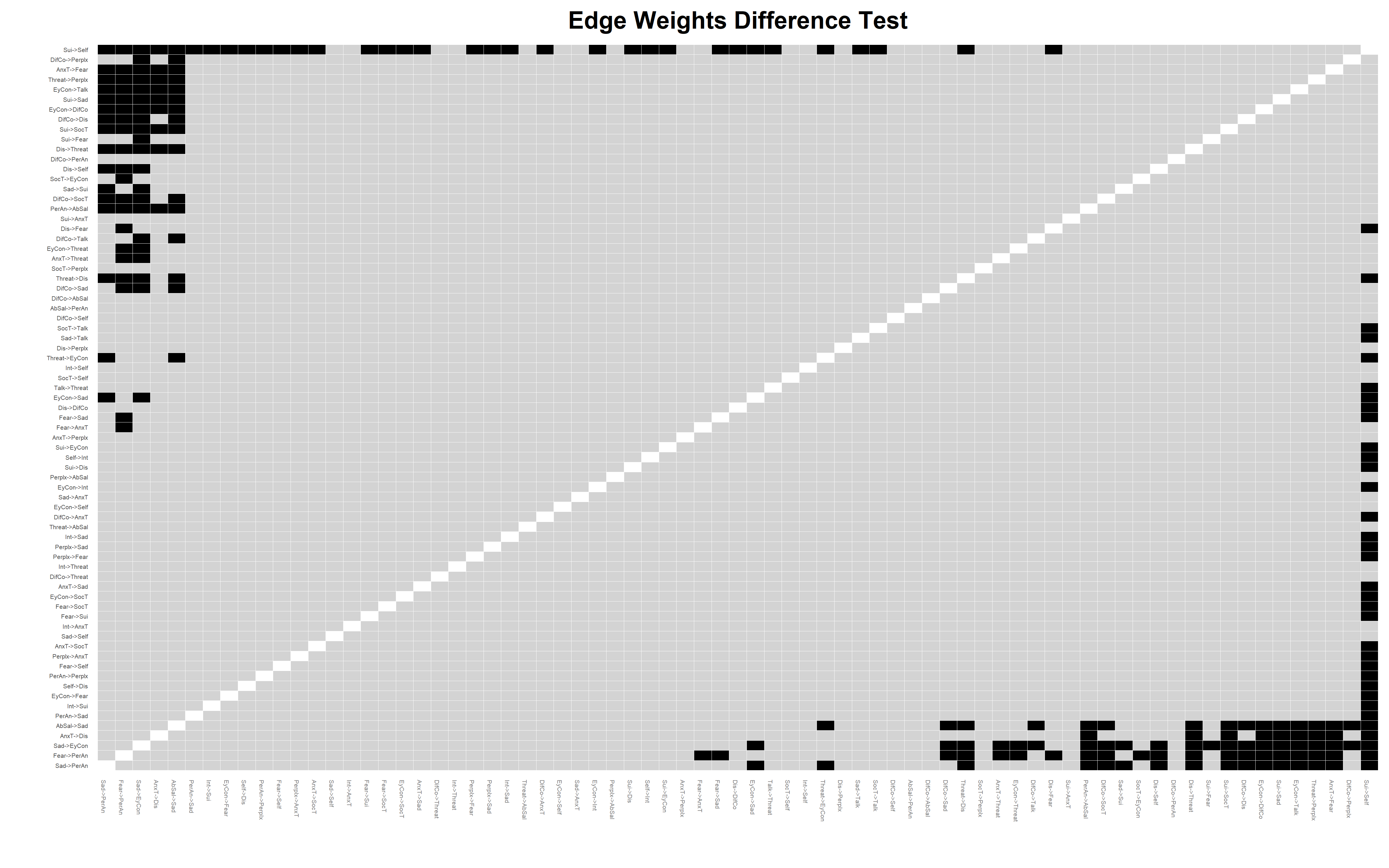


# **Figure S2.** Difference tests of edges

Rows and columns represent the different edges in the network, with edges ordered from most negative weight (left, bottom) to most positive (right, top). Black boxes indicate that two edges differ from each other at the α = 0.05 level.

**
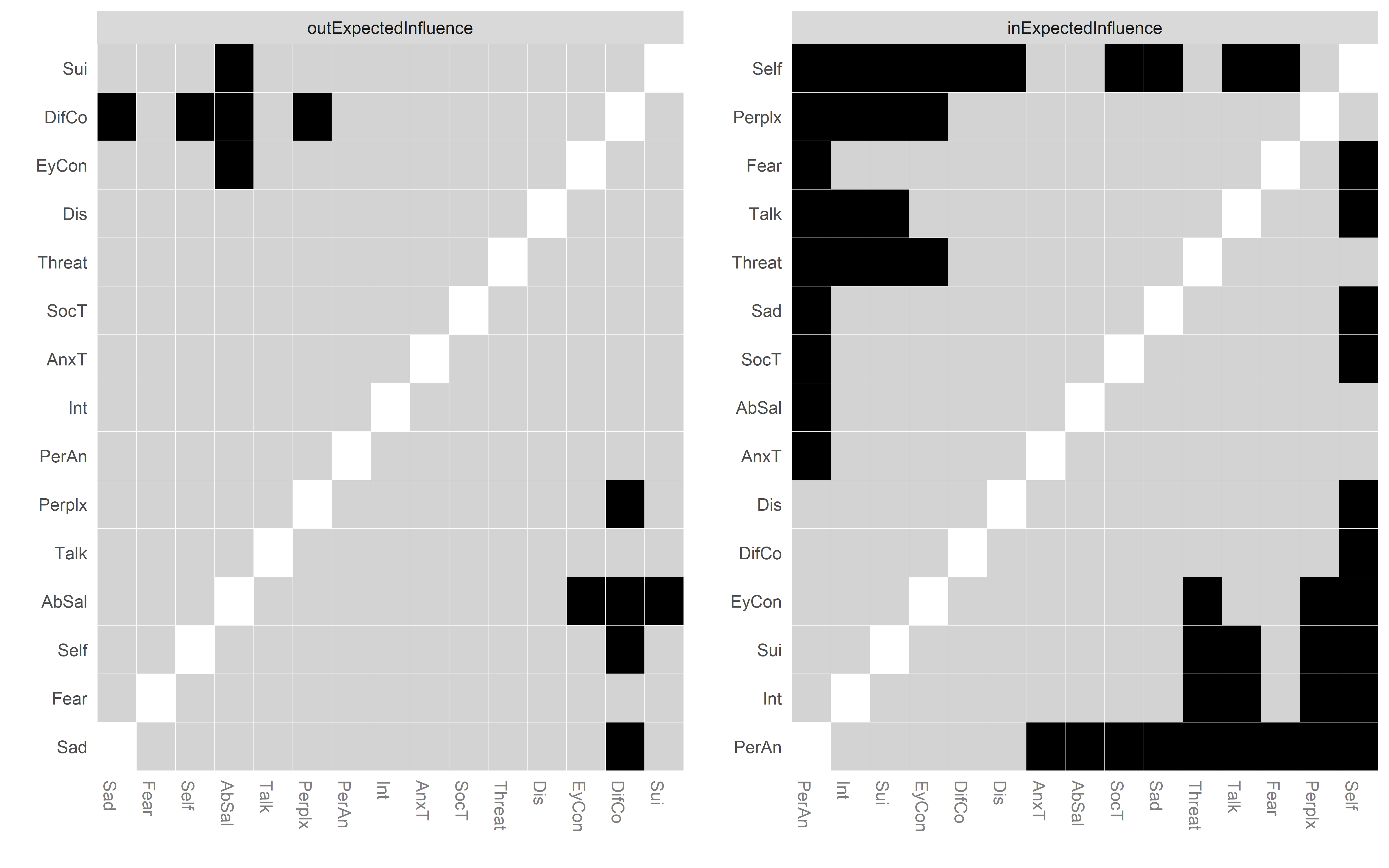
**

# **Figure S3.** Difference tests of centralities

Rows and columns represent the nodes in the network. Black boxes represent significant differences at the α = 0.05 level.


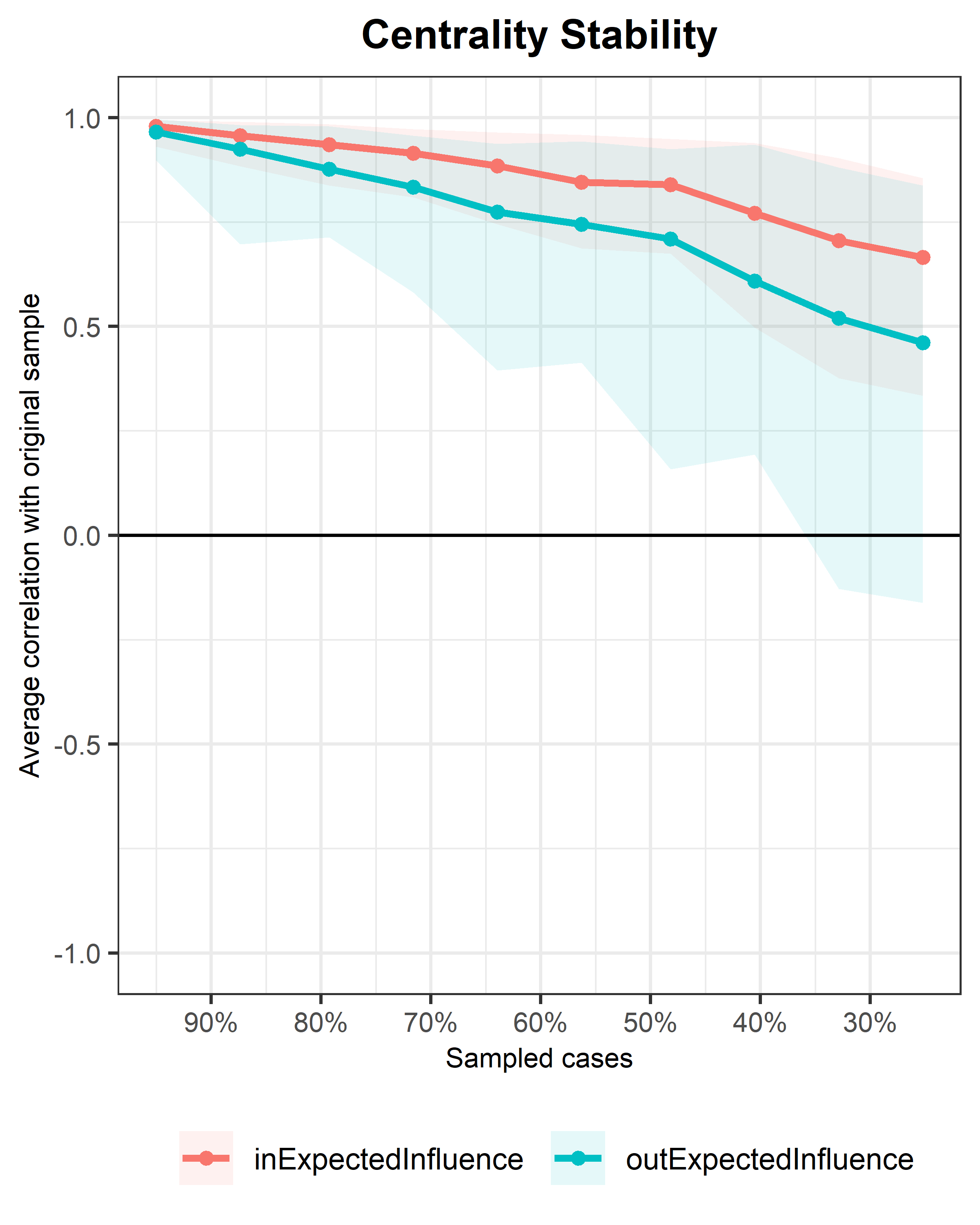


# **Figure S4.** Stability of the centrality measures

The x-axis shows the percentage of patients that was dropped. The y-axis shows the correlation of the centralities after dropping to the original centralities. The shaded areas indicate the 95% confidence interval.


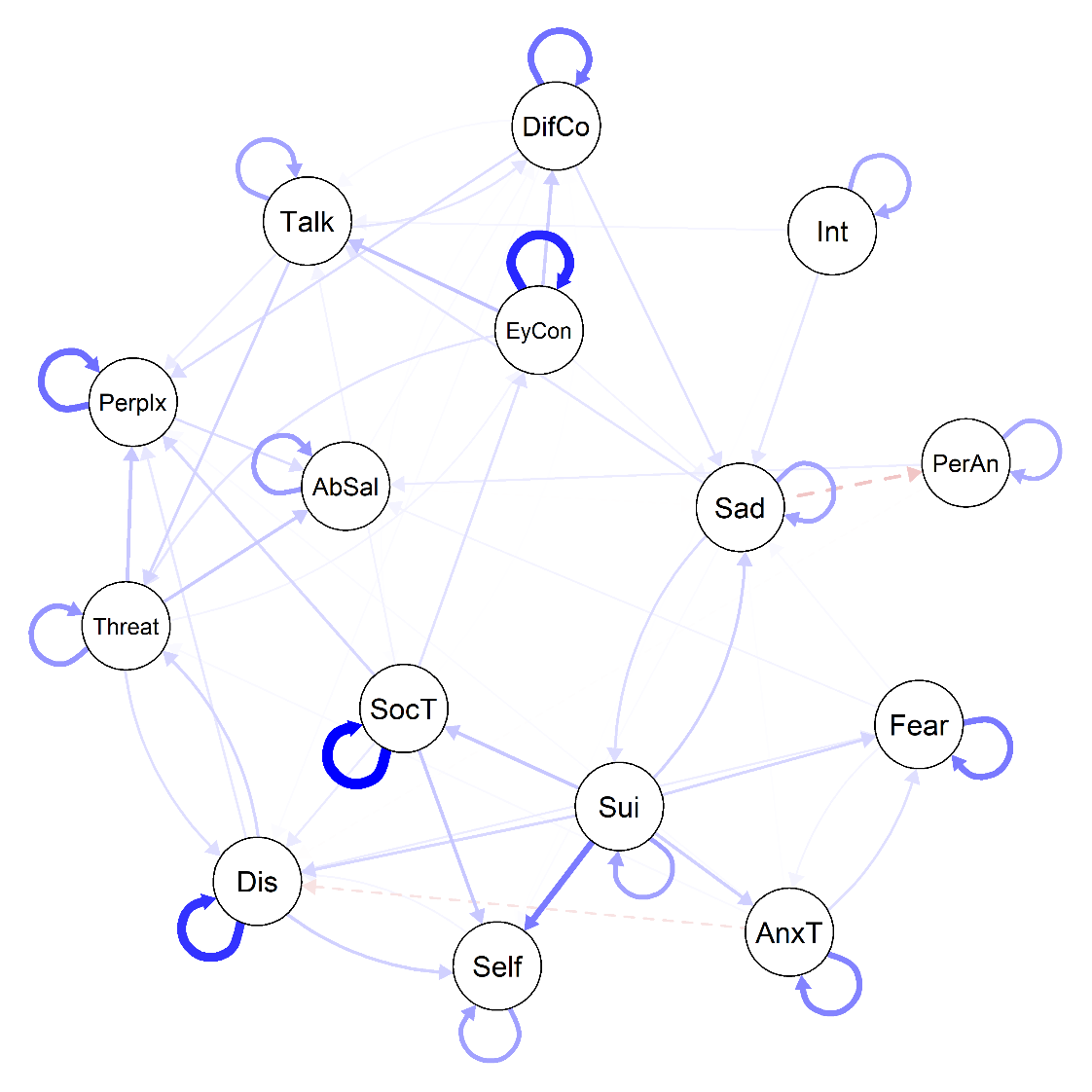


# **Figure S5**. Control cross-lagged network without misophonia with autoregressive effects. Nodes represent the variables included in the network and edges with arrows indicate a directed association between nodes. Solid edges represent positive associations while dashed edges represent negative associations.

Abbreviations: AbSal = aberrant salience, AnxT = anxious tension, DifCo = difficulty mixing with co-workers, Talk = difficulty talking with others, Dis = difficulty disagreeing, EyCon = difficulty making eye contact, Fear = fears, Int = interest, PerAn = perceptual anomalies, Perplx = perplexity; lack of natural evidence, Sad = sad, Self = self-view, Sui = suicidal ideation, SocT = social tension, Threat = feeling threatened; paranoia.


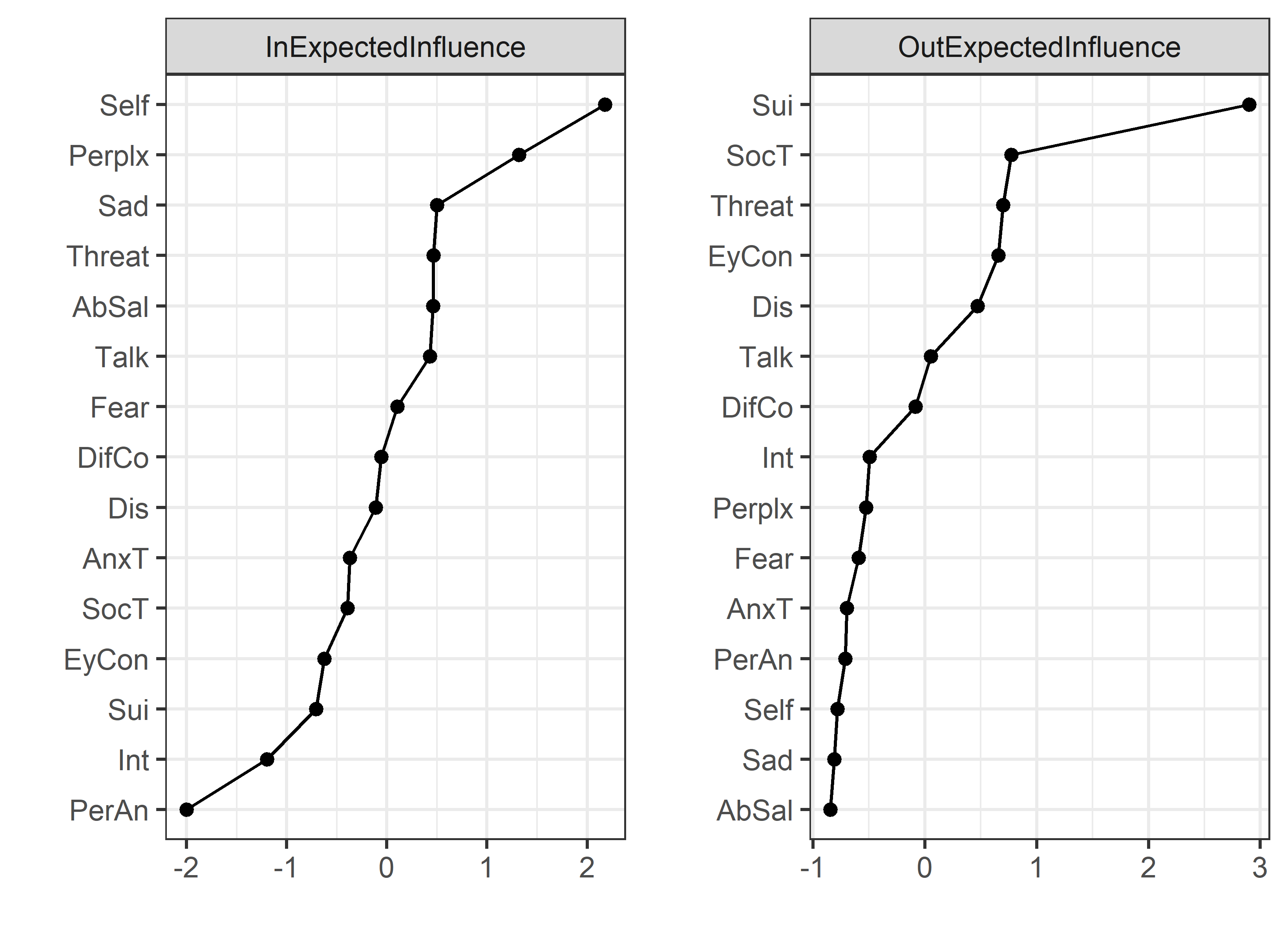


# **Figure S6**. Cross-lagged centrality plots for control network without misophonia.

The nodes are denoted on the y-axis and the standardized centrality coefficients are denoted on the x-axis. Higher z-scores indicate higher centrality.
Abbreviations: AbSal = aberrant salience, AnxT = anxious tension, DifCo = difficulty mixing with co-workers, Talk = difficulty talking with others, Dis = difficulty disagreeing, EyCon = difficulty making eye contact, Fear = fears, Int = interest, PerAn = perceptual anomalies, Perplx = perplexity; lack of natural evidence, Sad = sad, Self = self-view, Sui = suicidal ideation, SocT = social tension, Threat = feeling threatened; paranoia.

**
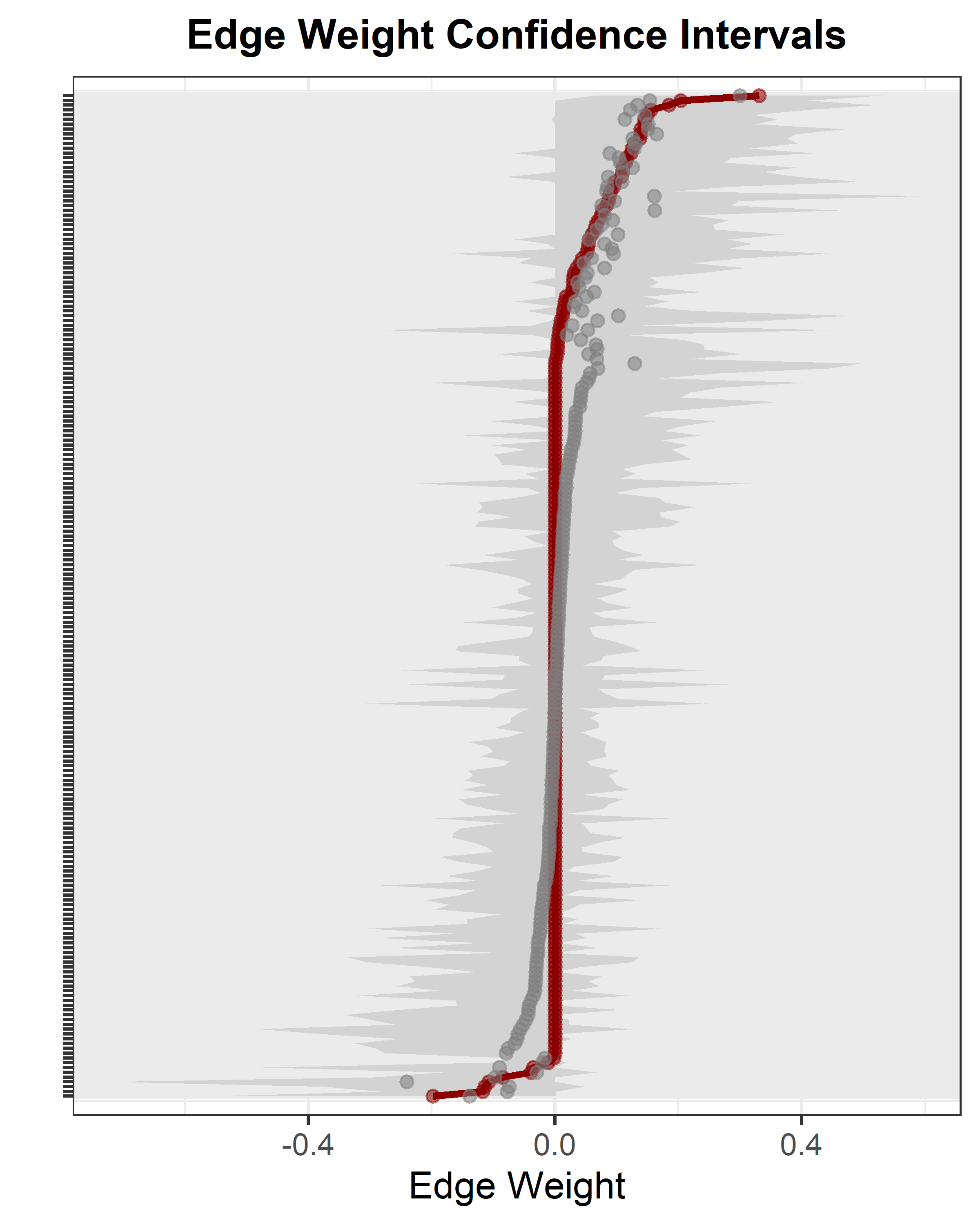
**

# **Figure S7**. Accuracy of the estimated edges for control network without misophonia

The x-axis shows the strength of the edge. The edges from the original network are shown in red and are arranged from most negative to most positive along the y-axis. The grey area represents confidence intervals based on the bootstrapped networks.


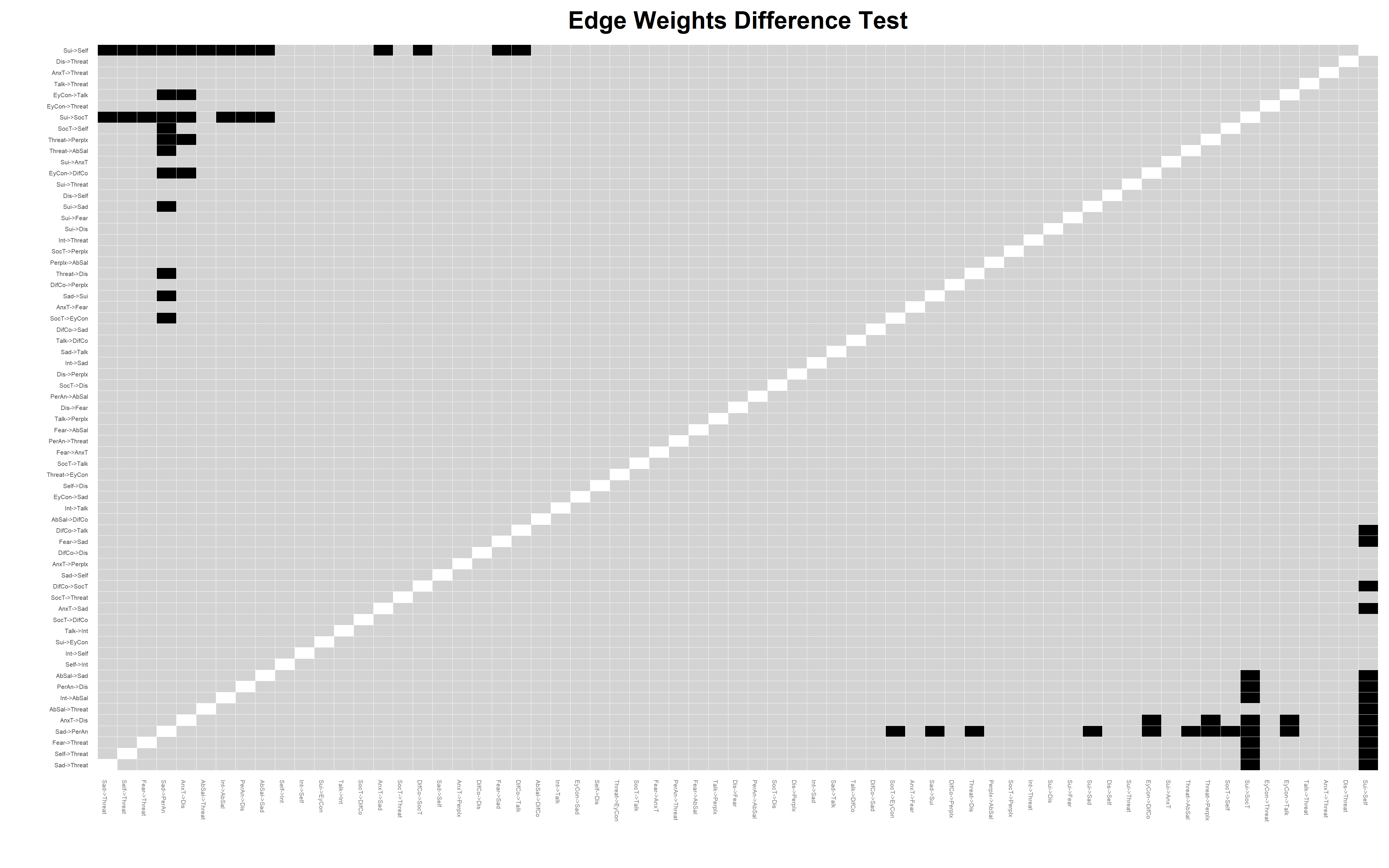


# **Figure S8.** Difference tests of edges for control network without misophonia

Rows and columns represent the different edges in the network, with edges ordered from most negative weight (left, bottom) to most positive (right, top). Black boxes indicate that two edges differ from each other at the α = 0.05 level.


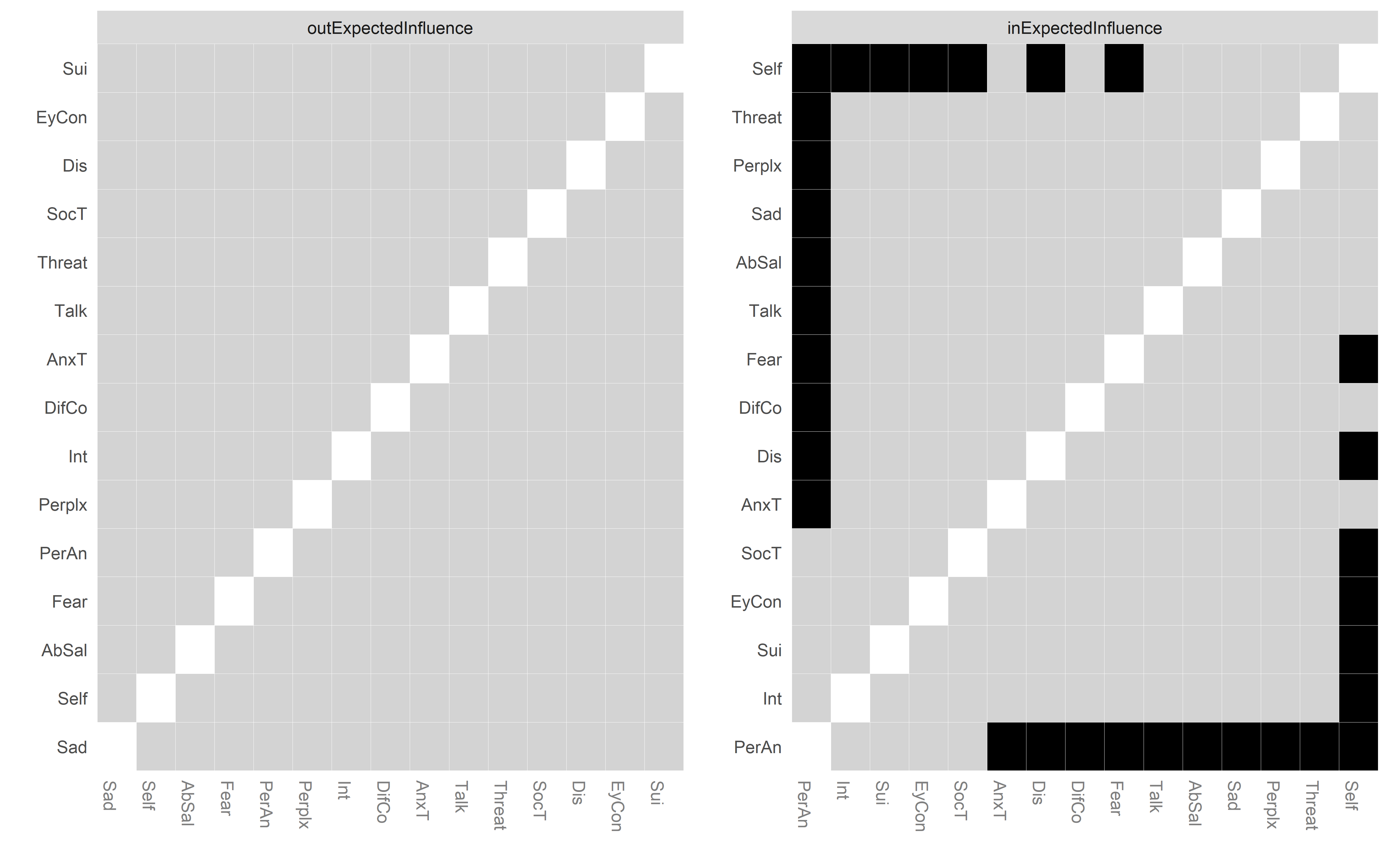


# **Figure S9.** Difference tests of centralities for control network without misophonia

Rows and columns represent the nodes in the network. Black boxes represent significant differences at the α = 0.05 level.
Abbreviations: AbSal = aberrant salience, AnxT = anxious tension, DifCo = difficulty mixing with co-workers, Talk = difficulty talking with others, Dis = difficulty disagreeing, EyCon = difficulty making eye contact, Fear = fears, Int = interest, PerAn = perceptual anomalies, Perplx = perplexity; lack of natural evidence, Sad = sad, Self = self-view, Sui = suicidality, SocT = social tension, Threat = feeling threatened; paranoia.


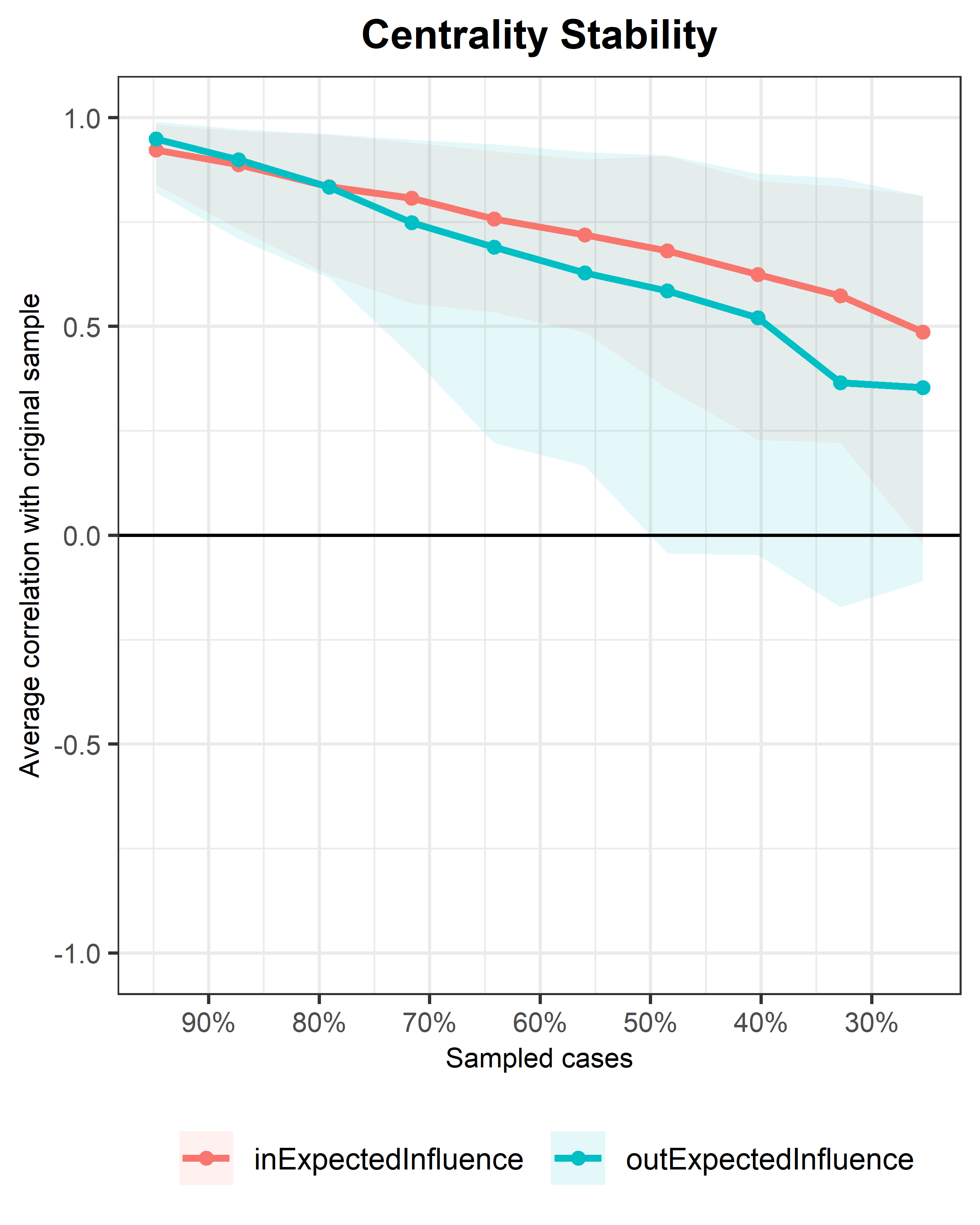


# **Figure S10.** Stability of the centrality measures for control network without misophonia

The x-axis shows the percentage of patients that was dropped. The y-axis shows the correlation of the centralities after dropping to the original centralities. The shaded areas indicate the 95% confidence interval.

**References**

1. Rhemtulla M, van Bork R, Cramer A. Cross-lagged network models. *Multivariate Behav Res*. 2020.

2. Rush AJ, Trivedi MH, Ibrahim HM, Carmody TJ, Arnow B, Klein DN, et al. The 16-item quick inventory of depressive symptomatology (qids), clinician rating (qids-c), and self-report (qids-sr): A psychometric evaluation in patients with chronic major depression. *Biol Psychiatry*. 2003;54(5):573-83.

3. Peters L, Sunderland M, Andrews G, Rapee RM, Mattick RP. Development of a short form social interaction anxiety (sias) and social phobia scale (sps) using nonparametric item response theory: The sias-6 and the sps-6. *Psychol Assess*. 2012;24(1):66.

4. Bech P. Clinical psychometrics: John Wiley & Sons; 2012.

5. Golino H, Christensen A, Moulder R. Eganet: Exploratory graph analysis: A framework for estimating the number of dimensions in multivariate data using network psychometrics. *R package version 09*. 2020;2.

6. Kuipers J, Moffa G, Kuipers E, Freeman D, Bebbington P. Links between psychotic and neurotic symptoms in the general population: An analysis of longitudinal british national survey data using directed acyclic graphs. *Psychol Med*. 2019;49(3):388-95.

7. Ben-Zeev D, Young MA, Depp CA. Real-time predictors of suicidal ideation: Mobile assessment of hospitalized depressed patients. *Psychiatry Res*. 2012;197(1):55-59.

8. Thompson WK, Gershon A, O'Hara R, Bernert RA, Depp CA. The prediction of study-emergent suicidal ideation in bipolar disorder: A pilot study using ecological momentary assessment data. *Bipolar Disord*. 2014;16(7):669-77.

9. Yamokoski CA, Scheel KR, Rogers JR. The role of affect in suicidal thoughts and behaviors. *Suicide Life Threat Behav*. 2011;41(2):160-70.

10. Moreno-Küstner B, Jones R, Švab I, Maaroos H, Xavier M, Geerlings M, et al. Suicidality in primary care patients who present with sadness and anhedonia: A prospective european study. *BMC Psychiatry*. 2016;16(1):94.

11. Michail M, Birchwood M. Social anxiety disorder in first-episode psychosis: Incidence, phenomenology and relationship with paranoia. *Br J Psychiatry*. 2009;195(3):234-41.

12. Combs DR, Penn DL. The role of subclinical paranoia on social perception and behavior. *Schizophr Res*. 2004;69(1):93-104.

13. Newman Taylor K, Stopa L. The fear of others: A pilot study of social anxiety processes in paranoia. *Behav Cogn Psychother*. 2013;41(1):66-88.

14. Reno RR, Kenny DA. Effects of self-consciousness and social anxiety on self-disclosure among unacquainted individuals: An application of the social relations model. *J Pers*. 1992;60(1):79-94.

15. Orr EMJ, Moscovitch DA. Blending in at the cost of losing oneself: Dishonest self-disclosure erodes self-concept clarity in social anxiety. *Journal of Experimental Psychopathology*. 2015;6(3):278-96.

16. Chambless DL, Hunter K, Jackson A. Social anxiety and assertiveness: A comparison of the correlations in phobic and college student samples. *Behav Res Ther*. 1982;20(4):403-04.

17. Swee MB, Butler RM, Ross BV, Horenstein A, O’Day EB, Heimberg RG. Interpersonal patterns in social anxiety disorder: Predictors and outcomes of cognitive-behavioral therapy. *Cognit Ther Res*. 2021;45(4):614-27.

18. Kapur S. Psychosis as a state of aberrant salience: A framework linking biology, phenomenology, and pharmacology in schizophrenia. *Am J Psychiatry*. 2003;160(1):13-23.

19. Wright A, Nelson B, Fowler D, Greenwood K. Perceptual biases and metacognition and their association with anomalous self experiences in first episode psychosis. *Conscious Cogn*. 2020;77:102847.

20. Cicero DC, Docherty AR, Becker TM, Martin EA, Kerns JG. Aberrant salience, self-concept clarity, and interview-rated psychotic-like experiences. *Journal of Personality Disorders*. 2015;29(1):79-99.
